# Dendritic Cell-Derived Exosomes Promote Natural Killer Cell Activation and Proliferation: A Role for NKG2D Ligands and IL-15Rα

**DOI:** 10.1371/journal.pone.0004942

**Published:** 2009-03-25

**Authors:** Sophie Viaud, Magali Terme, Caroline Flament, Julien Taieb, Fabrice André, Sophie Novault, Bernard Escudier, Caroline Robert, Sophie Caillat-Zucman, Thomas Tursz, Laurence Zitvogel, Nathalie Chaput

**Affiliations:** 1 Institut National de la Santé et de la Recherche Médicale, Unité 805, Villejuif, France; 2 Institut Gustave Roussy, Villejuif, France; 3 Center of Clinical Investigations in Biotherapies CICBT507, Institut Gustave Roussy, Villejuif, France; 4 Department of Hepatogastroenterology, Hôpital Européen Georges Pompidou, APHP, Paris, France; 5 Department of Immunotherapy, Institut Gustave Roussy, Villejuif, France; 6 Department of Dermatology, Institut Gustave Roussy, Villejuif, France; 7 Institut National de la Santé et de la Recherche Médicale, Unité 561, Hôpital Saint Vincent de Paul, Paris, France; 8 Faculté de Médecine de l'université Paris-Sud XI, Le Kremlin-Bicêtre, France; University of Sheffield, United Kingdom

## Abstract

Dendritic cell (DC) derived-exosomes (Dex) are nanomeric vesicles harboring functional MHC/peptide complexes promoting T cell-dependent tumor rejection. In the first Phase I trial using peptide-pulsed Dex, the observation of clinical regressions in the absence of T cell responses prompted the search for alternate effector mechanisms. Mouse studies unraveled the bioactivity of Dex on NK cells. Indeed, Dex promoted an IL-15Rα- and NKG2D-dependent NK cell proliferation and activation respectively, resulting in anti-metastatic effects mediated by NK1.1^+^ cells. In humans, Dex express functional IL-15Rα which allow proliferation and IFNγ secretion by NK cells. In contrast to immature DC, human Dex harbor NKG2D ligands on their surface leading to a direct engagement of NKG2D and NK cell activation *ex vivo*. In our phase I clinical trial, we highlight the capacity of Dex based-vaccines to restore the number and NKG2D-dependent function of NK cells in 7/14 patients. Altogether, these data provide a mechanistic explanation on how Dex may stimulate non MHC restricted-anti-tumor effectors and induce tumor regression *in vivo*.

## Introduction

A growing body of evidence shows that a variety of solid human tumors are spontaneously infiltrated by T cells and that memory effector T cells are associated with a favorable clinical outcome while overwhelming regulatory T cells markedly compromise long term survival [1]–[4]. This past decade paved the way to the conceptual basis of therapeutic vaccines against cancer, whereby the induction of tumor antigen-specific T cell immunity would lead to tumor eradication [5], [6]. The molecular characterization of cytotoxic T cell (CTL) defined-epitopes [7]–[9] and the anti-tumor effects promoted by adoptive transfer of tumor antigen-specific CTL confirmed the role of T cell immunity in the control of cancer growth at least in melanoma bearing patients [10], [11]. Indeed, this strategy can lead to more effective clinical responses against metastatic diseases when the adoptive transfer of tumor infiltrating T cells (TIL) is associated to lymphodepleting or myeloablative regimen [10], [12], [13]. Efficient vaccination against established tumors have been described in a variety of mouse tumor models, guiding clinical protocols in humans but the numerous approaches e.g. peptides, DNA or viral vaccines have thus far met with little success in the clinic because additional adjuvants are needed [14], [15]. The dendritic cells (DC), nature's adjuvants, represent essential component of any type of vaccination strategy [16], [17]. Preclinical studies have shown that, whenever tested, DC can be superior to other vaccine designs [18]. The immunogenicity of antigens delivered on DC has now been demonstrated in healthy human volunteers [19]. A number of clinical trials have utilized tumor antigen-loaded DC as vaccines in humans and some clinical and immune responses without toxicity have been observed [20], [21]. Nevertheless, the response rate using immune vaccine averages 3 to 17% [22], [23]. A difficult task of immunotherapy protocols is the identification of immunological parameters predicting clinical benefit. While the ultimate goal should be long term survival, such an endpoint requires large patient enrolment and long term follow up. Therefore, there is a need for surrogate markers predictive of clinical outcome. However, in most vaccinated patients, even in those who displayed tumor regression, it has been difficult to ascertain the existence of anti-vaccine T cell responses [24]–[27]. Surrogate markers of efficacy could be the detection of antigen spreading i.e. the identification of tumor specific CTL recognizing epitopes that were not present in the initial vaccine [28], [29], suggesting that alternate effectors elicited by the vaccine could mediate tumor destruction and subsequent CTL responses [30]. Natural killer (NK) cell activation can be critical to link innate and cognate immune responses [31]–[33]. Numerous adjuvants including DC can prime NK cell functions *in vivo* [34]–[37]. So far, investigators have overlooked the follow up of NK cell responses in clinical settings of discrepancy between tumor regressions and T cell responses.

DC derived-exosomes (Dex) have been originally shown to eradicate tumors in a T cell- dependent and Major Histocompatibility Complex (MHC)-restricted manner [38]. We demonstrated that Dex could transfer functional MHC class I and class II/peptide complexes to DCs leading to the priming of CD8^+^ and CD4^+^ T cells respectively [39]–[41]. The two first clinical trials assessing the feasibility and safety of Dex pulsed with MHC class I and class II tumor peptides in metastatic melanoma [42] and lung cancer patients [43] revealed that large amounts of exosomal MHC class II molecules can be purified in good manufacturing conditions (GMP) from autologous immature DC cultures to allow safe and prolonged immunization with Dex. If occasional tumor regressions were also observed in these studies, no DTH responses, peptide dose dependent effects nor T cell responses could be detected [42], [43]. Thus, we postulated that Dex could induce anti-tumor responses by acting on other MHC unrestricted immune effector cells. In these Phase I trials, we reported NK cell triggering in half of the patients following Dex vaccination [42]–[44]. However, the mechanisms accounting for the Dex bioactivity on NK cells remained unclear. Here we show that Dex can directly trigger NK cell activation in humans and mice, with a critical role for membrane bound Natural Killer Group 2 member D (NKG2D) ligands and IL-15Rα in this bioactivity. Moreover, the first Phase I clinical study revealed that Dex vaccines enhanced NK cell numbers and NKG2D-dependent functions in the majority of patients.

## Results

### Mouse Dex promoted NK cell proliferation and activation *in vivo*

**Figure 1:**
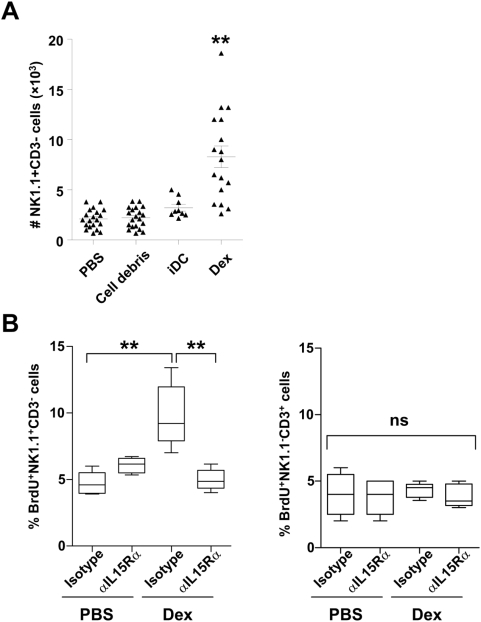
Mouse Dex promoted NK cell proliferation: a role for IL-15Rα. A. *Dex induce NK cell influx in draining lymph nodes*. Enumeration of CD3^−^ NK1.1^+^ cells (NK cells) in the draining lymph node following intradermal inoculation of 10 µg of mouse Dex, or 3×10^5^ immature DC (iDC), or 10 µg of irrelevant pelleted proteins (cell debris) or PBS. B. *NK cells enter cell cycle following Dex inoculation*. Proportion of BrdU^+^ CD3^−^ NK1.1^+^ cells (NK cells) (left panel) or BrdU^+^ CD3^+^ NK1.1^−^ cells (T cells) (right panel) in the draining lymph node following intradermal inoculation of 10 µg of PBS or mouse Dex in the presence of anti-IL-15Rα blocking mAb (αIL-15Rα) or isotype control mAb (Isotype). The graphs depict the means of absolute numbers or percentages±SEM of the data from 4 pooled experiments.* p<0.05, ** p<0.01 and ns: “non significant”.

To investigate the effects of Dex on NK cells *in vivo*, we inoculated intradermally 10 µg of exosomal proteins produced from bone marrow-derived DC (BM-DC). Thirty six hours later, we observed a 4–5 fold increase in the number of CD3^−^NK1.1^+^ NK cells in the draining lymph node (Fig. 1A). In contrast immature DC (iDC), from which Dex were derived, or irrelevant protein pellets (cell debris) obtained during Dex purification, failed to induce a rise in NK cell number (Fig. 1A). To assign the Dex-mediated NK cell accumulation to recruitment or active proliferation, we injected bromodeoxyuridine (BrdU) after Dex administration *in vivo*. BrdU was significantly and selectively incorporated in NK cells supporting the proliferative role of Dex on NK cells (Fig. 1B, left panel). Neither CD3^+^ T cells (Fig. 1B right panel) nor B cells (not shown) entered cellular division following Dex administration.

IL-2 and trans-presentation of IL-15 by IL-15Rα is required for NK cell survival, homeostasis and proliferation [45], [46], [47]. Consequently, we investigated the role of IL-15Rα and IL-2 in the Dex-mediated NK cell proliferation *in vivo*. As shown in Figure 1B, anti-IL-15Rα neutralizing mAb abolished NK cell proliferation (measured by BrdU incorporation) in the draining lymph node while isotype matched control antibodies failed to do so. Blocking IL-2 did not induce any change in the capacity of Dex to induce NK cell proliferation and activation (data not shown).

**Figure 2:**
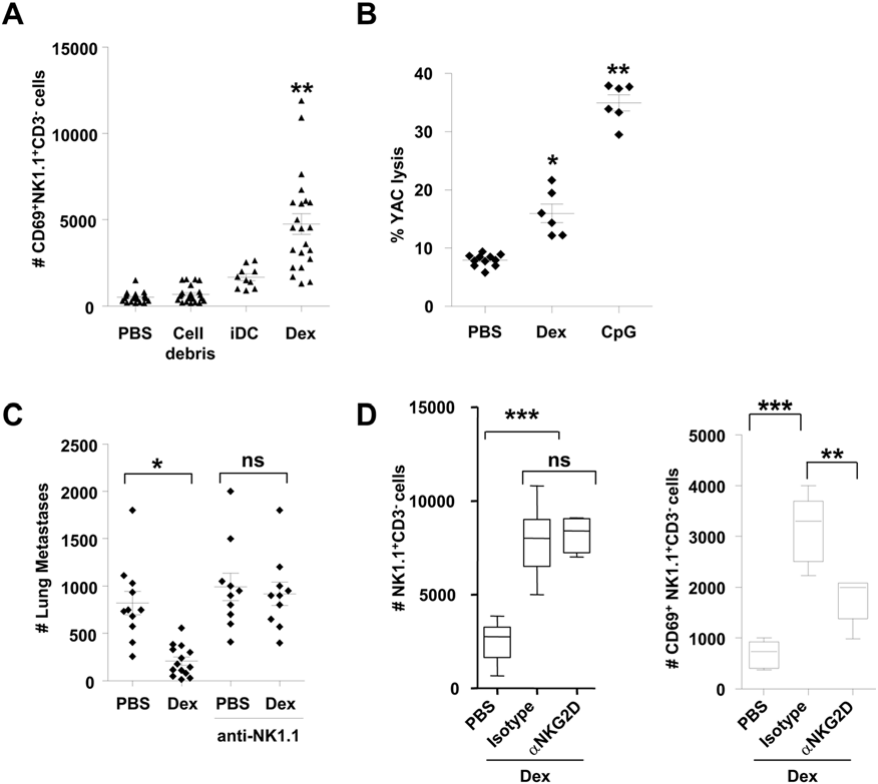
Mouse Dex promoted NK cell activation: a role for NKG2D. A. *Dex induced NK cell activation in the draining lymph node*. Absolute numbers of CD3^−^ NK1.1^+^ CD69^+^ cells in the draining lymph node following inoculation of 10 µg of mouse Dex, or 3×10^5^ iDC or 10 µg of irrelevant pelleted proteins (cell debris) or PBS. Each dot represents the result in one mouse. B. *Dex stimulated splenic NK cytotoxicity*. Killing assays on splenocytes against YAC-1 targets at 200∶1 and 50∶1 (not shown) after intradermal inoculations of 10 µg of Dex every other week for 8 weeks and sacrifice 48 hrs after the last immunization, or 24 hrs after a single subcutaneous injection of 10 µg of CpG ODN. C. *Therapy with unpulsed Dex reduced number of metastases*. Intradermal inoculation of 20 µg of exosomal proteins or PBS 5 days after intravenous injection of 3.10^5^ B16F10 tumor cells. Depletion of NK cells was achieved using 3 administrations of anti-NK1.1 mAbs or isotype control mAbs. Mice were sacrificed on day 10 for enumeration of lung metastases. D. *A role for NKG2D in Dex-mediated NK cell triggering*. Enumeration of CD3^−^NK1.1^+^ cells (left panel) and CD3^−^ NK1.1^+^CD69^+^ cells (right panel) in the draining lymph node following intradermal inoculation of PBS or 10 µg of mouse Dex in the presence of anti-NKG2D blocking mAb (αNKG2D) or control isotype mAb. The graphs depict the data of 4 pooled experiments (2 for B.). Means and SEM are shown. * p<0.05, ** p<0.01, *** p<0.001 and ns: “non significant”.

Administration of Dex not only induced NK cell proliferation but also activation, since NK cells up-regulated the CD69 activation marker in the draining lymph node (Fig. 2A). It is interesting to note that immature DC or cellular debris obtained during Dex purification failed to induce significant CD69 expression on NK cells (Fig. 2A). Moreover, NK cell cytotoxic activity was also triggered by Dex administration (Fig. 2B). The lytic activity against YAC-1 cells of splenocytes harvested from mice which received 8 weekly injections of Dex was markedly enhanced compared to controls receiving PBS (Fig. 2B). Consequently, a curative injection of Dex (at day 5) could significantly reduce the number of lung metastases in an NK1.1-dependent manner (Fig. 2C). These data clearly indicate that Dex can mediate NK cell activation *in vivo*.

NK cell triggering results from a balance between activating and inhibitory signals. Natural Killer Group 2 member D (NKG2D) is an activating receptor whose aberrant loss in cancer induces immune evasion [48]. NKG2D expressed by NK cells, CD8^+^ TCR (T cell receptor) αβ and γδT cells can overcome the inhibition imparted by MHC class I-specific inhibitory receptors [49]. In NK cells, NKG2D serves as a primary activation receptor, which by itself triggers cytotoxicity [50]. We investigated the role of NKG2D ligands in Dex-mediated NK cell activation by monitoring the numbers and activation status of NK cells in the draining lymph node in the presence of Dex and neutralizing anti-mouse NKG2D mAb. Although the accumulation of NK cells was not affected by NKG2D blockade (Fig. 2D, left panel), the numbers of CD3^−^NK1.1^+^ expressing CD69^+^ significantly dropped in the presence of anti-NKG2D mAb (Fig. 2D, right panel).

Altogether, Dex trigger an IL-15Rα and a NKG2D-dependent NK cell proliferation and activation respectively, in secondary lymphoid organs in mice.

### Human Dex harbour functional IL-15Rα and synergize with IL-15 for NK cell proliferation and IFNγ production *in vitro*

**Figure 3:**
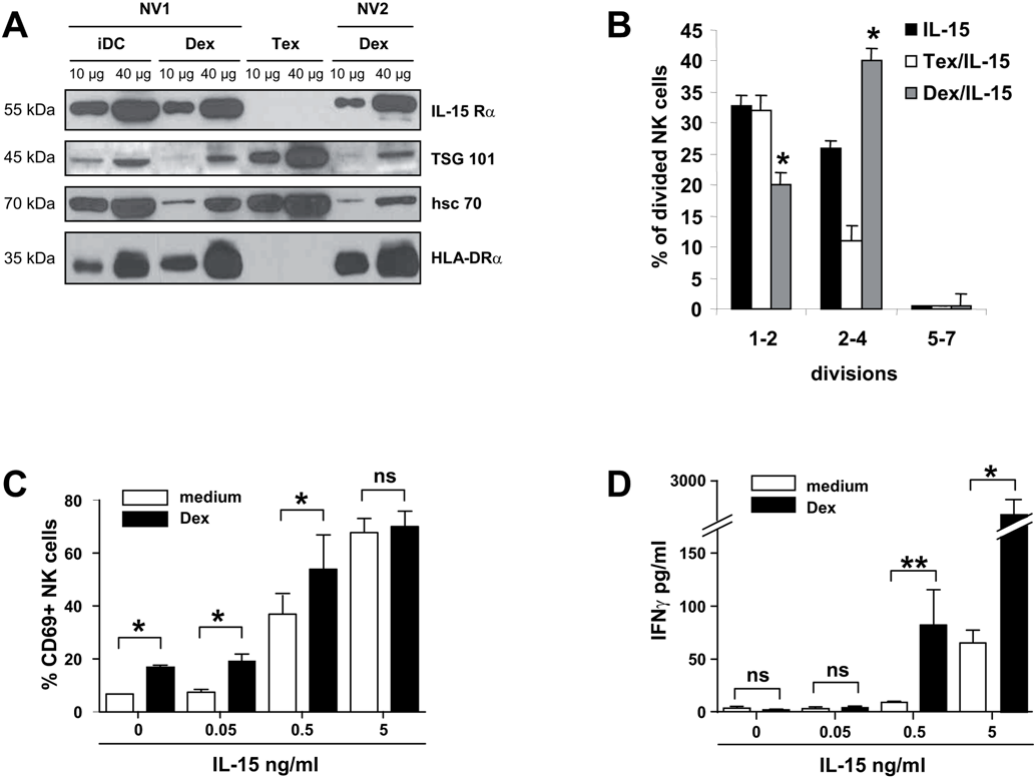
Human Dex harbour functional IL-15Rα and synergize with IL-15 for NK cell proliferation *in vitro* and IFNγ production *in vitro.* *A.* *Immunoblotting of IL-15Rα from Dex and DC lysates*. Western Blot analysis on 10–40 µg of protein lysates obtained from immature DC (iDC), Dex or Tex (exosomes from Mel888 melanoma cell line) using anti-IL-15Rα mAb. Positive controls included anti-HLA-DRα, -TSG 101 and -HSC 70 Abs. Representative immunoblots of two normal volunteers are depicted (NV1 and NV2). Molecular weights are indicated on the left lane. B. *Proliferative effects of recombinant IL-15 and Dex on NK cells*. CFSE-labeled NK cells were cultured with or without 10 µg autologous Dex or allogenic Tex in complete medium containing 0.5 ng/ml of human recombinant IL-15. At day 6 of culture, NK cell proliferation was determined by flow cytometry and the number of divisions were counted and depicted. A representative experiment out of two is shown. C–D. *Synergistic effects between Dex and recombinant IL-15 for NK cell triggering*. NV's PBL were cultured without (white histograms) or with (black histograms) 10 µg autologous Dex and increasing concentrations of human recombinant IL-15. NK (CD56^+^ CD3^−^) cells were then analysed for CD69 expression by flow cytometry (C) or supernatants were harvested to measure IFNγ levels in EIA (D). The graphs depict means±SEM of % of CD69 expressing NK cells in 3 experiments (C) or IFNγ concentrations in 4 experiments (D). * p<0.05, ** p<0.01 and ns: non significant.

We next assessed whether human Dex express IL-15Rα molecules. As shown in Figure 3, IL-15Rα was detected in immunoblotting of several human Dex preparations purified from immature DC obtained from normal volunteers (NV) (Fig. 3A). Other cytokine receptors such as IL-2Rα and IL-7Rα could not be detected on Dex preparations (data not shown). However, IL-15 was not observed in similar conditions (not shown). In contrast, tumor-derived exosomes (Tex) harvested in the supernatants of melanoma cell lines did not bear IL-15Rα molecules (Fig. 3A). Since Dex harbored IL-15Rα but not IL-15, we next assessed whether Dex could *trans-present* exogenous IL-15 to NK cells. As shown in Figure 3B, autologous Dex (but not Tex) enhanced the IL-15-driven proliferation of NK cells. As IL-15/IL-15Rα present on DC induced NK cell activation [51], we studied the activation status of NK cells stimulated with Dex and/or rhIL-15. Dex as well as rhIL-15 alone could induce CD69 expression on NK cells (Fig. 3C). When rhIL-15 and Dex were combined, only an additive effect could be observed regarding CD69 expression on NK cells (Fig. 3C). On the other hand, no production of IFNγ could be measured when NK cells were stimulated by Dex alone. However, the combination of Dex with rhIL-15 could significantly enhance IFNγ secretion by NK cells (Fig. 3D) indicating that Dex and rhIL-15 could synergize to induce IFNγ production by NK cells *in vitro*.

These data highlight that IL-15Rα harbored by Dex is functional, leading to NK cell proliferation and activation *in vitro* when associated with rhIL-15.

### Human Dex bear NKG2D ligands leading to engagement of NKG2D receptors

**Figure 4:**
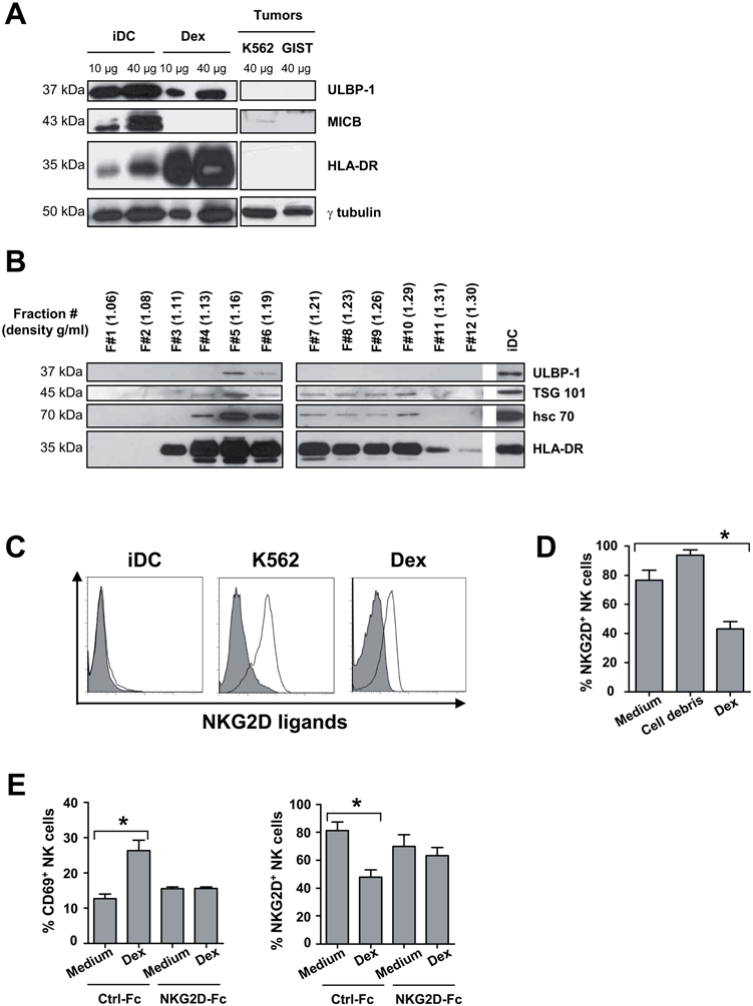
Human Dex harbour functional NKG2D ligands. A–B. *Immunoblot detection of NKG2D ligands on Dex*. (A) 10–40 µg of protein lysates from immature DC (iDC), Dex or tumor cells (K562, GIST) were assayed in western blot analyses using anti-ULBP-1 and MICB Abs on whole protein lysates (A) (this result was similar in 6 Dex preparations from 6 different healthy donors) or on each fraction of distinct density (B) following ultracentrifugation of 400 µg of Dex proteins on a continuous density gradient. Controls included anti-HLA-DRα, TSG 101, hsc 70 Abs and anti-γ tubulin Abs are also depicted. Molecular weights are indicated on the left lane. Note that the density of the ULBP-1 positive Dex fractions is approximately 1.16 to 1.19 g/ml i.e. the assumed density flotation of Dex. C. *Dex express cell surface NKG2D ligands*. Flow cytometry analyses of the surface expression of NKG2D ligands (empty histograms) on beads coated-Dex using rhNKG2D-Fc chimera or a mix of anti-human MICA/B, ULBP-1, ULBP-2 and ULBP-3 mAbs and appropriate secondary Abs. Similar stainings of iDC and K562 cells (a negative and positive control respectively). Filled histograms represent stainings with isotype matched mAbs. D. *Engagement of NKG2D receptors on NK cells triggered by Dex*. NK cells were incubated 40 hrs with medium or 10 µg autologous Dex or irrelevant pelleted proteins (cell debris) coated onto MaxiSorp™ wells, and then stained with anti-CD3 APC, anti-CD56 CyC, anti-NKG2D PE mAb. Flow cytometry analyses revealed the mean % (±SEM) of NKG2D expressing NK cells in three independent experiments performed in triplicate wells. E. *NKG2D-dependent NK cell activation by Dex in vitro*. Identical setting as in D. but using rhNKG2D-Fc fusion proteins or Ig-Fc controls to determine CD69 (left panel) and NKG2D (right panel) expression on NK cells in flow cytometry analyses. * p<0.05.

In mouse models, Dex induced NK cell activation in an NKG2D-dependent manner. Next, we evaluated the presence of NKG2D ligands (NKG2D-L) on human Dex by immunoblotting and flow cytometry. Western blot analyses revealed the presence of ULBP-1, one of the NKG2D-L, in Dex preparations obtained from normal volunteers (Fig. 4A). ULBP-1 was also detected in DC lysates. However, MICB present in DC was not identified in Dex (Fig. 4A). We could systematically detect ULBP-1 in normal volunteers' Dex preparations whereas no MICA/B could be found in 6 independent experiments. To rule out any possible contaminants, we performed extensive purification of Dex on a continuous sucrose density gradient and confirmed the presence of ULBP-1 at density gradient corresponding to Dex floatation i.e. 1.16 to 1.19 g/ml (Fig. 4B). Flow cytometry analyses on Dex coupled to microbeads confirmed the presence of NKG2D ligands at the surface of Dex (Fig. 4C). In contrast, iDC failed to expose cell surface NKG2D-L (Fig. 4C). Following coculture of purified NK cells with autologous Dex, NK cells could i) up-regulate CD69 and HLA-DR (Fig. 3C and not shown) and ii) selectively downregulate their surface expression of NKG2D receptors (Fig. 4D), suggesting that NKG2D-L harboured on Dex were functional. Indeed, the levels of NKp46 remained stable on NK cells exposed to Dex (not shown). These two effects could be prevented by pre-incubation of Dex with recombinant NKG2D-Fc fusion proteins that block the interaction of NKG2D receptors with their ligands (Fig. 4E).

Altogether, as for mouse Dex, human Dex can activate NK cells through a NKG2D-dependent mechanism.

### Dex vaccination restored numbers and NKG2D-dependent functions of NK cells in advanced melanoma patients

We have previously reported the feasability and safety of vaccination with Dex pulsed with MAGE3.A1 and MAGE3.DP04 peptides in 15 HLA-A1/B35+ and DP04+ stage IIIb and IV melanoma patients [42]. In this phase I trial, CD4^+^ or CD8^+^ T cell responses specific for the vaccinating peptides could not be detected [42]. Because Dex could induce tumor regressions in 4 melanoma patients but could not elicit T cell activation directed neither against the vaccinating epitopes nor against autologous tumor cells [42], we postulated that NK cells could account for tumor shrinking.

**Figure 5:**
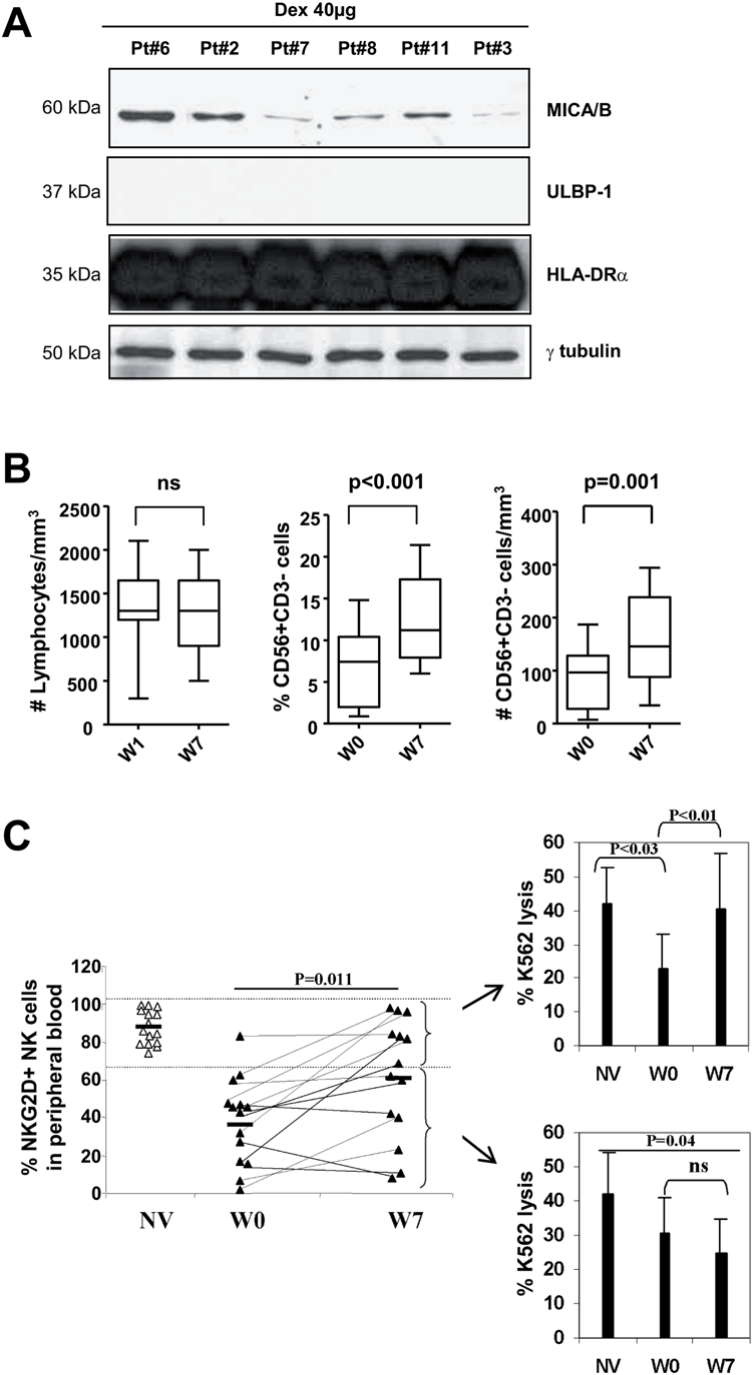
Vaccination of melanoma patients with Dex restored NKG2D-dependent NK cell function. A. *Patients' Dex harbour MICA/B molecules*. Western Blot immunodetection of NKG2D-L using anti-MICA/B and anti- ULBP-1 Abs (not detected) on 40 µg of Dex proteins. B. *Dex enhanced the numbers of circulating NK cells in melanoma patients*. Enumeration of lymphocytes (left panel) and flow cytometry determination of the percentages (middle panel) and absolute numbers (right panel) of CD3^−^ CD56^+^NK cells in PBMC prior to (W0) and following Dex vaccination (W7) in the Phase I trial enrolling 14 melanoma patients. C. *Restoration of NKG2D expression and function by Dex therapy in melanoma*. Flow cytometry analyses of NK cells using anti-NKG2D or isotype matched control mAb, anti-CD3, and anti-CD56 mAbs were performed on PBL of normal volunteers (NV) or of patients before Dex therapy (W0) and after Dex therapy (W7). Purified autologous NK cells from 14 patients enrolled in the Phase I trial at W1 or W7 or from 10 NV were investigated for cytotoxic activity against ^51^Cr labeled K562 cells at a 10∶1 (shown) and 2∶1 (not shown) E∶K562 ratio. Two tests per individual were run yielding identical results. Intra-individual variations were <10%. Means±SD for 10 normal volunteers (NV), for 14 melanoma patients before Dex therapy (W0) and after Dex therapy (W7) are represented. The upper panel depicts the NK cell cytotoxicity when NKG2D expression levels were restored at W7 (>70%) in contrast to the lower panel indicating the NK cell cytotoxicity in patients whose NKG2D expression remained low (<70%). * p<0.05 and ns: non significant.

Dex obtained from patients also harboured NKG2D-L as shown in immunoblotting (Fig. 5A). Contrary to NV Dex, patients' Dex express MICA/B and not ULBP1 (Fig. 5A). Although NKG2D ligands on Dex were different between NV and melanoma patients, we could not observe any functional consequences regarding NKG2D-dependent NK cell activation *in vitro* (data not shown). Fifteen patients received 4 vaccines at weekly intervals and were assessed for T and NK cell functions before (W1) and 7 weeks (W7) after the first Dex inoculation. While the lymphocyte pool remained stable throughout Dex therapy, the proportion and the absolute number of circulating CD3^−^/CD56^+^ NK cells/mm^3^ significantly increased after 4 weekly vaccinations with Dex (Fig. 5B). The study of the NK cell phenotype in these advanced melanoma patients revealed that the levels of NKG2D were profoundly decreased compared with normal volunteers prior to Dex vaccination (Fig. 5C) (36±22%) but significantly rose (p = 0.011) after 4 injections of Dex vaccines to 61±28% (Fig. 5C). To assess the functional relevance of this observation, we tested the killing activity of blood NK cells against K562, a NKG2D ligand-expressing target (Fig. 4C), before and after Dex therapy in the two cohorts of patients i.e. those who normalized their NKG2D expression levels and those who did not (Fig. 5C). Seven out of 14 patients recovered expression levels of NKG2D ≥70% after 4 Dex vaccines. Among these responders, the depressed K562-specific cytotoxicity reverted back to normal levels after Dex inoculation (Fig. 5C, upper panel). However, non-responders (i.e. patients in whom Dex did not restore NKG2D expression) exhibited comparatively low killing activity against K562 (Fig. 5C, lower panel). In two patients exhibiting tumor regression (pt#3, pt#12) and continuing on treatment (Dex administration every other three weeks for 6–10 months), the NK cell effector functions remained boosted at later time points (W30) (data not shown). Interestingly, Dex therapy could also up-regulates NKG2D expression on CD8^+^ T cells in 6/14 patients tested (data not shown).

These data support the notion that Dex inoculation in patients enhanced the proportion and absolute number of circulating NK cells and restored NKG2D expression levels on circulating T and NK cells, thus stimulating the MHC unrestricted NKG2D-dependent cytotoxicity.

## Discussion

Here, we provide the first evidence that GMP manufactured dendritic cell-derived exosomes can trigger NK cell proliferation and activation *in vitro* and in patients. Although other reports claimed that exosomes harboring the nuclear factor BAT3 [52] (NKp30-interacting partner) or HSP70 derived-peptides [53], [54] could induce NK cell triggering, our data focusing on DC derived-exosomes in mice and humans demonstrate a critical role for IL-15Rα-and NKG2D for Dex-mediated NK cell proliferation and activation respectively.

Designing of DC vaccines inducing an efficient NK cell response could aid the establishment of Th1 polarization of cognate immune responses. However, NK cell activation in vaccination protocols has been rarely studied [32]. *Ex vivo* protocols generating DC capable of secreting high levels of IL-12 (TLR3 ligands and type 1 IFN or CD40L/agonistic CD40 mAb, IFNγ, IL-1β containing regimen, *ex vivo* licensing by activated NK cells) are currently developed [55], [56]. Osada and colleagues were the first to monitor NK cell responses in tumor patients vaccinated with DC. They showed that NK cell responses following DC vaccination may correlate more closely with clinical outcome than do T cell responses [55]. Other cell-free vaccines containing heat shock proteins derived from melanoma or colon tumors could induce NK cell activation in patients and may contribute to the priming of T cell responses and/or tumor shrinking [57]. In our clinical trial aimed at vaccinating with Dex melanoma patients, we have been able to show that Dex boosted recirculation of NK cells and restored NKG2D expression and function on NK cells.

Upon binding to its ligand, NKG2D stimulates the NK cell lytic pathway, resulting in killing of transformed or altered cells. NKG2D engagement induced the secretion of cytokines such as IFNγ and GM-CSF [58]. However, Oppenheim et al. have demonstrated that long term exposure to NKG2D ligands resulted in impaired natural cytotoxicity *in vivo* and reduced tumor immunosurveillance [59]. Indeed, NK or CD8^+^ T cells from cancer patients often exhibit a profound downregulation of the expression of NKG2D receptors. The NKG2D downregulation in cancer patients has been correlated with high levels of circulating MICA/B molecules shed by the tumor burden [60], or with circulating mature TGF-β or to regulatory T cells expressing membrane bound TGF-β [60]–[62]. We were not able to detect significant concentrations of plasma TGF-β or serum MICA/B in our 14 melanoma patients that could account for the low expression of NKG2D receptors (not shown). Here we describe a novel form of shed NKG2D ligands which is membrane bound and harbored by Dex. Indeed, Dex convey functional NKG2D ligands both in mice and humans leading to NK cell triggering. This phenomenon contrasts with shed NKG2D ligands that are inhibitory [59].

Different teams have reported that tumor-derived exosomes (Tex) could modulate NK cell activity. *In vitro*, Tex have been described to enhance NK cell function through Heat Shock Protein 70 (Hsp70) surface expression [53]. These Hsp70 positive Tex have been shown to synergize with NKG2D ligands expressed on the tumor cell surface resulting in reduction of tumor growth and suppression of metastatic disease [54]. However, Liu and colleagues have suggested that Tex could inhibit NK cells through the blockade of IL-2 mediated NK cell activation leading to tumor escape [63]. Finally, Clayton and colleagues have shown that Tex could harbor MICB molecules leading to down regulation of NKG2D on peripheral blood lymphocytes *in vitro* (CD8^+^ T cells and CD3^+^CD8^−^ T cells) [64]. This diminution of NKG2D expression on CD8^+^ T cells was associated with an inhibition of T cell cytotoxicity *in vitro*. Consequently, authors claimed that NKG2D expression on Tex could be another mechanism of tumor invasion [64]. More recently the same team described that Tex-induced NKG2D down-modulation was partially due to NKG2D ligands expressed on Tex [65]. In contrast they clearly showed that this effect was the consequence of TGF-β1 carried by Tex [65]. For Dex few works have shown their capacity to activate NK cells *in vitro* [52]. In our study dealing with Dex, we clearly show that Dex could engage NKG2D on NK cells *in vitro*, a signaling cascade that might synergize with IL-15Rα triggering. Moreover our data provide for the first time the demonstration that the level of NKG2D expression on NK cells (Fig. 5) and CD8^+^ T cells (not shown) are increased after 4 vaccinations with Dex in humans. Indeed, the presence of other receptors such as IL-15Rα may contribute to restore the level of NKG2D expression on NK and CD8^+^ T cells allowing restoration of these lymphocyte functions at least *ex vivo* (Fig. 5C and not shown). This observation demonstrates that the composition of exosomes is critical for NK cell activation since Tex that do not harbor IL-15Rα (Fig. 3A) failed to do so. Consequently in association with recombinant IL-15 Dex were able to boost IFNγ secretion (Fig. 3D). In contrast, Tex can decrease effector function of NK cells. IL-15, a recognized potent positive modulator of NKG2D-dependent responses of NK cells is rendered poorly activating in the presence of Tex [65]. This observation that exosomal NKG2D ligands release can lead to NK activation highlight a novel mechanism of NKG2D dependent NK cell triggering. Indeed, while exosomal forms of NKG2D ligands seem to activate NK cells, the release of soluble forms that are mediated through shedding activity inhibits NK cell functions.

*Trans*-presentation of IL-15 as been described to have a pivotal role in NK cell homeostasis, survival and proliferation [46], [66], [67]. Moreover, it can enhance and restore NKG2D expression and function [68]. Dex harbored functional IL-15Rα (Fig. 3) but lacked IL-15 (data not shown). Since IL-15 is produced by a vast diversity of cells *in vivo*, we can postulate that endogenous IL-15 is presented by IL-15Rα harbored by Dex leading to significant NK cell proliferation *in vivo* after Dex vaccination (Fig. 3) and NKG2D restoration in melanoma patients (Fig. 5). A recent work showed that *in vivo* delivery of IL-15/IL-15Rα complexes triggers rapid and significant regression of established solid tumors in two murine models [69]. These data provide novel insights into the use of IL-15/IL-15Rα complexes to relieve tumor-resident immune cells from functional suppression by the tumor microenvironment and have significant implications for cancer immunotherapy [69]. Dubois and coll. also demonstrated that mimicking IL-15 *trans*-presentation strongly increased the IL-15-mediated proliferation of murine NK and CD8^+^/CD44^high^ T cells. When mice bearing the NK-sensitive syngeneic tumor B16 were treated, the presence of IL-15Rα-IgG1-Fc increased the anti-tumor activity of IL-15 [70]. Thus, Dex could represent a new tool for IL-15 *trans*-presentation *in vivo*.

Dex express both IL-15Rα and NKG2D ligands that can synergize to induce NK cell triggering. Therefore, Dex could represent a link between innate and antigen specific T cell responses that could participate in tumor regression [33], [71]. We indeed showed that in the absence of Treg [72] or in the presence of TLR agonists [41], Dex could trigger tumor antigen-specific CD8^+^ T cell responses leading to tumor regression [73]. Knowing that NK cell recruitment/proliferation and activation play a key role in promoting Th1 differentiation [71], Dex could represent a suitable vaccine that could activate in unison innate and adaptive immunity. Engineering Dex with increased expression of both IL-15Rα and NKG2D ligands could lead to a significant enhancement of anti-tumor responses *in vivo*. Different works have demonstrated that the maturation stage of DC that produce Dex have a critical impact on the Dex composition leading to a better capacity to activate T and NK cells [52], [74]–[76]. In our lab, our preliminary unpublished data demonstrate that Dex produced from IFNγ-treated DC are enriched with NKG2D ligands and IL-15Rα. This observation remains to be confirmed and the functional relevance of this phenomenon has to be demonstrated. In consequence, we are currently, in Gustave Roussy Institute, launching a phase II clinical trial in which metronomic dosage of cyclophosphamide followed by Dex vaccination (these Dex are produced from mature DC and harbor IL-15Rα and NKG2D ligands) are evaluated for the treatment of inoperable patients bearing stage IIIB/IV non-small cell lung cancer who responded or experienced disease stabilization after the first line chemotherapy. During this clinical trial, we will monitor both immune functions in vaccinated patients and Dex phenotype to determine if the regulation of the specific sorting of NKG2D-L in Dex appears to be also different between NV and non-small cell lung cancer patients. We hope to reinforce the postulate that NK cell together with T cell responses might cooperate to correlate with clinical responses.

## Materials and Methods

### Protocol design, patients' characteristics and eligibility criteria

The study was approved by the Kremlin Bicêtre Hospital Ethics Committee (Comité de Protection des Personnes) and regulatory authorities, and informed written consent was obtained from each patient. Fifteen patients bearing melanoma fulfilling the inclusion criteria were enrolled in the study. Patients received a 4 week vaccination course with antigen loaded Dendritic cell-derived exosomes (Dex) given intradermal and subcutaneous injections every week for a total of 4 vaccinations as already described [42].

### Mice

Female C57BL/6 (H-2^b^) wild type (BL6) were obtained from the “Centre d' Elevage Janvier” (Le Genest St Isle, France), and maintained in IGR animal facilities according to the Animal Experimental Ethics Committee Guidelines.

### DC culture

Mouse bone marrow derived-DC (BM-DC) were cultured as previously described [41]. Briefly, bone marrow progenitor cells were grown in IMDM culture medium (Sigma-Aldrich, France) supplemented with 50 U/ml penicillin, 50 µg/ml streptomycin, 2 mM L-glutamine, 10% decomplemented fetal calf serum (Gibco-BRL, France), 50 µM 2-ME (Sigma-Aldrich) and 30% J558-mGM-CSF culture supernatants for 10–12 days. Human monocyte-derived DC (MD-DC) were generated from normal volunteers' monocytes purified after elutriation of peripheral blood according to the French EFS procedures (Pr J. Bernard, Institut Jean Godinot, Reims, France). MD-DC were cultured in bags under adherence-free conditions (Lifecell, Baxter) for 5 days in serum-free AIMV medium (Gibco-BRL) supplemented with 1000 UI/ml of rhGM-CSF (R&D Systems) and 200 UI/ml of rhIL-4 (Schering Plough). Patients' monocyte-derived DC (MD-DC) were obtained according to a good manufacturing process already described [77]. MD-DC were generated from the adherent fraction of peripheral blood mononuclear cells (PBMC) in AIM-V medium (Gibco-BRL) supplemented with 1000 IU/ml of rhu GM-CSF (Leucomax, Schering-Plough, Levallois-Perret, France) and 1000 IU/ml of rhu IL-4 (Schering Plough, Kenilsworth, NJ) as previously described [78].

### Exosome production and purification from mice and human DC

Mouse Dex were manufactured using the same procedure as that described for the MELADEX clinical trial [77]. For exosome production, cells were cultured for 48 h to 72 h in complete medium depleted from FCS-derived exosomes by diafiltration. Exosomes were isolated following a process of ultrafiltration/diafiltration described by Lamparski and colleagues [77]. The supernatant of the resulting dendritic cell preparation was harvested, filtered, subjected to serial centrifugation to remove cells and debris, and ultrafiltered through a 500-kDa filter. This preparation was underlaid with 30% sucrose/D2O and ultracentrifuged at 100,000×g. The cushion containing exosomes was diafiltrated to remove the sucrose. Exosomes were pelleted by ultracentrifugation at 100,000×g, resuspended in PBS and stored at −80°C. In patients, exosomes were purified from MD-DC culture supernatants according to a good manufacturing process already described [42], [77].

### Sucrose gradient

Purified exosomes were resuspended in 2 ml of Hepes 20 mM / Sucrose 2.5 M and floated into an overlaid linear sucrose gradient (2–0.25 M) in a SW41 tube for 16 hrs at 100,000×g. Gradient fractions of 1 ml were collected from top to bottom and analysed by SDS-PAGE and immunoblotting.

### *In vivo* assays *in mice*

**Determination of NK cell number in LN.** Increasing amounts of Dex (up to 10 µg) and graduated numbers of immature DC (up to 3.10^5^ iDC) or 10 µg of irrelevant pelleted proteins were inoculated in one footpad of BL6 mice. Popliteal ipsi- and contro-lateral nodes (PBS injected) were harvested at 36 hrs. In neutralizing experiments, Dex were inoculated in footpads along with 10 µg of anti-mouse NKG2D (eBioscience, San Diego, USA) or anti-mouse IL-15Rα (R&D Systems) neutralizing mAbs or with the corresponding isotype Ab. Lymph node mononuclear cells were mechanically minced. Cells were enumerated using trypan blue exclusion prior to immunostaining with three color mAbs anti-CD3 FITC, anti-NK1.1 APC and anti-CD69 PE (BD Pharmingen). **BrdU incorporation.** For BrdU incorporation assays, 1.5 mg of BrdU solution was injected per mice i.p. the day of Dex inoculation. The day after, lymph node NK or T cells were analyzed in flow cytometry using NK1.1/CD3 staining. BrdU incorporation was revealed using APC anti-BrdU antibody according to BrdU Flow kit (BD Pharmingen) and analyzed by flow cytometry (FACSCalibur, BD). **Assessment of NK cell cytotoxic activity.** Twenty µg of Dex or 20 µl of PBS were inoculated each week for 8 weeks or 10 µg ODN CpG 24 hrs before sacrifice (provided by Dr. A. Carpentier, AP-HP Pitié Salpétrière, Paris, France) were injected intradermally in BL6 mice. Spleens were mechanically minced. Cells were enumerated using trypan blue and used as effector cells. Cytotoxicity of splenocytes was measured in a standard 4 hrs ^51^Cr-release assay using Na_2_^51^CrO_4_-labeled YAC-1 targets. Experiments were conducted in triplicates at various E∶T ratios (200∶1 and 50∶1). **Antitumor experiments.** 3.10^5^ B16F10 tumor cells were inoculated in the tail vein at day 0, Dex or controls were injected at day 5 and mice were sacrificed at day 10 for the enumeration of lung metastases. NK cell depletion was achieved using i.p. injections of 300 µg of anti-NK1.1 mAb (PK136) or isotype control Ab at day-4, day0 and day+4 as described elsewhere [79].

### Assessment of NK cell activity in normal volunteers

***In vitro*** **proliferation experiments.** Ten µg of Dex were coated onto sterile MaxiSorp™ plate (Nunc) at 4°C during 18 hrs. The next day, autologous purified NK cells and 0.5 or 5 ng/ml of rhIL-15 (R&D Systems) in AIMV medium (Gibco-BRL) were added. Human NK cells were purified from frozen elutriated peripheral blood lymphocytes (PBL) with EasySep™ human NK cell enrichment kit (StemCell Technologies, Vancouver) and labelled with 2.5 µM CFSE (Sigma-Aldrich). At day 6 of coculture, half of the medium was replaced with fresh medium supplemented with cytokine. Cells were collected at day 6 and 14 and analysed for proliferation by flow cytometry. **NK cell activation assay.** PBL were cocultured with 10 µg autologous Dex bound onto MaxiSorp™ plate for 2 days. IFNγ secretion was measured with BD OptEIA kit in the supernatants. Cells were harvested and stained for flow cytometry analysis with anti-CD3 FITC, anti-CD69 PE (BD Pharmingen) and anti-CD56 APC mAbs (Beckman Coulter) or anti-CD3 APC, anti-CD69 FITC (BD Pharmingen), anti-CD56 CyC (Beckman Coulter) and anti-NKG2D PE (R&D Systems) mAbs. In blocking experiments, Dex were pre-incubated or not with rhNKG2D-Fc chimera (R&D Systems) or human Ig-Fc chimera control (Alexis Biochemical).

### Assessment of NK cell activity in patients

PBMC were isolated by Ficoll density centrifugation (Ficoll-Paque™, Amersham Pharmacia Biotech AB, Uppsala, Sweden) from leukaphereses performed before (W1) and after Dex vaccines (W7) according to the institutional guidelines. Cells were enumerated using trypan blue exclusion test. Cells were analysed by flow cytometry using anti-CD45, CD3 and CD56 mAbs for determination of total lymphocytes and NK cells numbers and NK cells percentages. Purified (85–90% CD3^−^/CD56^+^) NK cells were obtained from PBL of W1, W7 and W30 using EasySep™ human NK cell enrichment kit (StemCell Technologies, Vancouver). NK cells prior to (W1) or following Dex vaccines (W7 and W30) were tested in Na_2_^51^CrO_4_ chromium release assays against K562 cells after 40 hrs stimulation with AIMV culture medium alone in 96 U bottom well plates. Experiments were conducted in triplicates at various E∶T ratios. For the determination of NKG2D expression on NK cells before (W1) and after Dex therapy (W7), cells were analysed by flow cytometry using mouse anti-NKG2D PE (R&D Systems), anti-CD56 CyC (Beckman Coulter), and anti-CD3 APC mAbs (BD Pharmingen).

### Immunoblotting

Cells or exosomal proteins were extracted in lysis buffer (50 mM Tris-HCl, pH 7.5, 250 mM NaCl, 0.1% NP-40, 10 mM Na3VO4, 5 mM DTT, protease inhibitor cocktail tablet (Complete, Mini, EDTA-free, Roche)) for 30 min at 4°C. Nuclei and cell debris were removed by centrifugation. Proteins in lysates were quantified by DC™Protein Assay (Bio-Rad Laboratories). Aliquots of protein extracts were solubilised in Laemmli loading buffer and resolved by 10% SDS-PAGE and transferred onto nitrocellulose or PVDF membranes (Bio-Rad Laboratories). Membranes were saturated for 1 hr and incubated with primary Abs for 1 hr at room temperature or over night at 4°C. Primary Abs were probed with the appropriated horseradish peroxidase-labelled secondary Abs (Southern Biotech) and detected by SuperSignal West Pico Chemiluminescent Substrate (Pierce). Primary Abs included: anti-IL15Rα, anti-ULBP-1, anti-MICB (R&D Systems), anti-TSG101, anti-MICA/B C-19 (Santa Cruz Biotechnology), anti-hsc 70 (Stressgen), anti-γ Tubulin (Sigma-Aldrich) and anti-HLA-DRα (kindly provided by P. Benaroche, Institut Curie, France).

### Exosome surface analysis

Purified exosomes were coupled to 4 µm aldehyde / sulphate latex beads (Molecular Probes) as described elsewhere [77] and immunostained. Abs included mouse isotype controls or human Ig-Fc chimera as a negative control for Fc chimeric protein (Alexis Biochemicals), mouse anti-human MICA/B, ULBP-1, ULBP-2 and ULBP-3 or rhNKG2D-Fc chimera (R&D Systems), and FITC-conjugated goat anti-mouse IgG Fc (Jackson ImmunoResearch) or PE-conjugated goat anti-human IgG Fc (Rockland). To confirm exosome binding to beads, anti-CD63 PE, anti-CD81 PE (BD Pharmingen) and anti-CD82 PE (Diaclone) mAbs were used as a control. Human DC and K562 cells were used respectively as a negative and a positive control of the NKG2D ligands staining. The exosome-bead complexes and cells were analysed on a FACSCalibur flow cytometer with the FlowJo analysis software.

### Statistical analyses

All results are expressed as means±standard error of the mean (SEM) or as ranges when appropriate. For two groups, normal distributions were compared by the unpaired or paired Student's t test when appropriate; non-normal samplings were compared using the Mann-Whitney's test or Wilcoxon matched paired test when appropriate. For more than two groups, analyses of variances were performed with the Kruskal-Wallis test. Statistical analyses were performed using Prism 5 software (GraphPad, San Diego, CA). P values of <0.05 were considered significant.
